# *In Vitro* Cytochrome P450 Formation of a Mono-Hydroxylated Metabolite of Zearalenone Exhibiting Estrogenic Activities: Possible Occurrence of This Metabolite *in Vivo*

**DOI:** 10.3390/ijms10041824

**Published:** 2009-04-21

**Authors:** Frederique Bravin, Radu C. Duca, Patrick Balaguer, Marcel Delaforge

**Affiliations:** 1 CEA Saclay, iBiTec-S, SB2SM and URA CNRS 2096, Gif sur Yvette Cedex F-91191, France; E-Mails: Frederique.bravin@gmail.com (F.B.); radu.duca@ibna.ro (R.F.D.); 2 IRCM, Institut de Recherche en Cancérologie de Montpellier, 34298, France; INSERM, U896, Montpellier 34298, France; Université Montpellier 1, Montpellier 34298, France; CRLC Val d’Aurelle Paul Lamarque, 34298, Montpellier, France; E-Mail: p.balaguer@valdorel.fnclcc.fr (P.B.)

**Keywords:** Cytochrome P450, zearalenone, hydroxy-metabolites, estrogenic activities, human

## Abstract

The mycoestrogen zearalenone (ZEN), as well as its reduced metabolites, which belong to the endocrine disruptor bio-molecule family, are substrates for various enzymes involved in steroid metabolism. In addition to its reduction by the steroid dehydrogenase pathway, ZEN also interacts with hepatic detoxification enzymes, which convert it into hydroxylated metabolites (OH-ZEN). Due to their structures to that of estradiol, ZEN and its derived metabolites bind to the estrogen receptors and are involved in endocrinal perturbations and are possibly associated with estrogen-dependent cancers. The primary aim of this present study was to identify the enzymatic cytochrome P450 isoforms responsible for the formation of the most abundant OH-ZEN. We thus studied its *in vitro* formation using hepatic microsomes in a range of animal model systems including man. OH-ZEN was also recovered in liver and urine of rats treated orally with ZEN. Finally we compared the activity of ZEN and its active metabolites (α-ZAL and OH-ZEN) on estrogen receptors using HeLa ER-α and ER-β reporter cell lines as reporters. OH-ZEN estrogenic activities were revealed to be limited and not as significant as those of ZEN or α-ZAL.

## 1. Introduction

**Figure 1.**
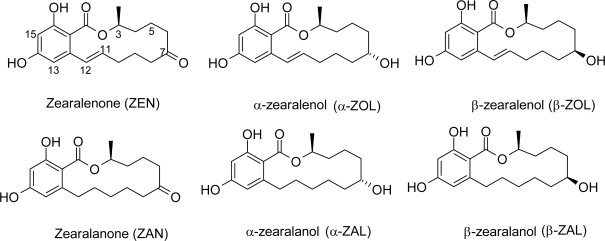
The chemical structures of zearalenone (ZEN), 7α-hydroxy-zearalenol (α-ZOL), 7β-hydroxy-zearalenol (β-ZOL) and their 11-12 reduced analogs: zearalanone (ZAN), 7α-hydroxy zearalanol (α-ZAL), 7β-hydroxy-zearalanol (β-ZAL).

The mycoestrogen zearalenone (ZEN) (Figure 1), a resorcyclic acid lactone ((3S,11*E*)-3,4,5,6,9,10-Hexahydro-14,16-dihydroxy-3-methyl-1H-2-benzoxacyclotetradecin-1,7(8*H*)-dione) produced by *Fusarium* strains, is found worldwide as a contaminant in cereals and grains, including maize and soybean [1, 2]. The ZEN derivatives α-zearalenol (α-ZOL), β-zearalenol (β-ZOL), α-zearalanol (α-ZAL), β-zearalanol (β-ZAL), zearalanone (ZAN), whose chemical structures are also shown in Figure 1, can also be detected in corn stems infected with *Fusarium* in the field [3] and in rice culture [4]. The relative ratio of the reduced ZEN metabolites, or the excretion of its glucuronide conjugates [see ref. 5 for a review], were found to be species dependent. In rats, most of the zearalenone was detected in urine as free zearalenone or glucuronide conjugate (catalyzed by UDPGT : uridine diphosphoglucuronyl transferases, [1]), while only the presence of small amounts of zearalenols (assumed to result from reduction by 3α- and 3β-hydroxysteroid dehydrogenases [5]) and their conjugates are produced [1, 6]. In fact, glucuronidation facilitates the pre-systemic elimination of hydrophobic toxins (and drugs); therefore, glucuronide conjugates of the parent compound and that of α-zearalenol predominates in most species. The main location of ZEN metabolism is in the liver [5, 6] however it is well understood that this is both species and organ specific (microsomal or post-mitochondrial) and includes the rates of formation of either α- or β-ZOL), [7].

Hence, considering the interaction with estrogen receptors and the modulation of steroid metabolism, ZEN is associated with compounds known collectively as endocrine disruptors. Previous *in vivo* and *in vitro* experiments have revealed that ZEN and its metabolites are substrates for various enzymes involved in steroid metabolism [8]. Despite its non-steroidal structure, ZEN resembles 17β-estradiol sufficiently to bind with strong affinity and activates estrogen receptors α and β (ER-α and ER-β) resulting in functional and morphological alterations in the reproductive organs [1,6,9].

ZEN is widely known to be enzymatically reduced to the α- and β-isomers of zearalenol [1,8-10]. ZEN also interacts with detoxification enzymes, which convert it into several unidentified hydroxylated metabolites, as evidenced recently by GC-MS analysis of liver microsomes [11]. Furthermore, ZEN has been found to enhance the expression of Cytochrome P450 (CYP3A) in cell cultures [12].

The aims of this present study were to identify the major form (OH-ZEN) of the ZEN hydroxylated hepatic metabolites produced *in vitro* in rat and from a range of different model animal species, including humans. Then we confirmed the cytochrome P450 involvement in metabolite formation and verified the *in vivo* occurrence of OH-ZEN in rats treated with ZEN. Finally we evaluated the ER-α and ER-β activities of OH-ZEN and compared it to that of ZEN and its more active metabolite α-ZAL using reporter cell lines [13].

## 2. Results and Discussion

### 2.1. In vitro ZEN metabolism in rat liver

**Figure 2.**
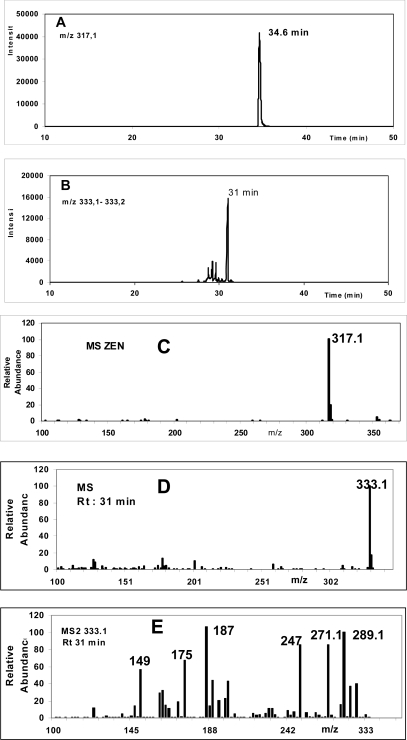
HPLC-MS chromatograms of A: m/z 317.1 (ZEN) and B: 333.1 (OH-ZEN), C: MS spectrum of ZEN, D: MS and E : MS2 spectrum of its OH-ZEN metabolite (Rt 31 min) formed after 30 minutes of *in vitro* incubation (1 μM total cytochrome P450 from PBtreated rat microsomes in presence of 50 μM ZEN and the NADPH-generating system, 0.1 M phosphate buffer pH 7.4).

Using incubations of phenobarbital (PB)-treated rat liver microsomes at pH 7.4 in the presence of NADPH, we observed the formation of a metabolite having a retention time (Rt 31 min, Figure 2A) different from those of ZEN (Rt 34.6 min, Figure 2B), or its reduced metabolites, α-ZOL and β-ZOL. This compound termed OH-ZEN has a *m/z* of 333.1, determined using ESI mass spectrometry in negative mode (Figure 2D), and corresponds to a mono-oxygenated metabolite of ZEN (M+16). The collision induced dissociation of the m/z 333.1 led to several fragments (Figure 2E) which allowed the precise location of the OH function on the 8 position of ZEN. For example, the fragment at m/z 247 arose from the loss of C1-C5 fragment or that at 187 and 149. Complete assignment of this hydroxylated metabolite is actually performed using several chromatography and MS techniques (Bravin *et al.* in preparation). This metabolite is formed in significant amounts using (PB) treated rat microsomes (about 5 nmoles/30 min/nmole P450) compared to untreated samples (1.7 nmoles/30 min/nmole P450). OH-ZEN is also found in post-mitochondrial incubation (fraction S9) in association with β-ZOL but not in the cytosolic fraction where only α- and β-ZOL were formed in the presence of NADPH. Additionally OH-ZEN can be detected in untreated or PB-treated rat hepatocytes incubated for 2 hours in the presence of 10 or 20 μM ZEN (data not shown).

The fact that OH-ZEN is only formed in microsomes in the presence of NADPH favors the involvement of cytochrome P450 in this process. This is reinforced by the fact that the formation of OH-ZEN is inhibited by carbon monoxide or by the presence of compounds having a high affinity for cytochrome P450 heme (2μM ketoconazole as an example).

It should be noted that other hydroxylated metabolites have been formed, but only the major OH-ZEN metabolite, α- and β-ZOL have been studied in the present work.

### 2.2. Cytochrome P450 isoforms involvement

#### 2.2.1. Expressed P450 isoforms

As incubations of ZEN with human, or phenobarbital-treated rat, microsomes led to the formation of OH-ZEN, we performed incubations with different human P450 isoforms and in particular families 2 and 3, that are known to be induced by PB [14] (Table 1).

Only human P450 isoforms CYP2C8, 3A4 and 3A5 led to significant quantities of OH-ZEN (more than 2 nmoles/nmole P450 in 60 min). These results were confirmed using rat expressed CYP2C isoforms. In this case CYP2C11 exhibit similar activity as of H CYP2C8, H CYP3A4 and H CYP 3A5 whereas R CYP2C6 and R CYP 2C13 exhibit lower activities. This is in agreement with the large variability of substrate recognition of the CYP2C family, as already mentioned [15,16].

### 2.3. In vitro ZEN metabolism by different species

In order to determine the presence and distribution of the OH-ZEN metabolite in other species, incubations under identical conditions with liver microsomal preparations from a range of different model systems were undertaken (Table 2). Microsomes from almost all the species investigated, including man, produced low amounts of OH-ZEN and its relative abundance compared with the other metabolites α-ZOL and β-ZOL was found to be species dependent. The greatest formation of OH-ZEN was obtained using dog and monkey microsomes.

Under the same conditions, PB-treated rat liver microsomes were at least 10 times more active than human female liver microsomes. Human male liver microsomes led to a non quantifiable amount of OH-ZEN.

The well known metabolic pathway of ZEN is its reduction to α– or β-ZOL *via* steroid dehydrogenases. In addition to this reduction pathway, it has been proposed that hydroxylation can occur *in vitro* not only on the aliphatic part of the molecule but also on it aromatic locus [17].

It is an interesting fact that in the *in vitro* studies mouse and pig do not form significant amounts of OH-ZEN, whereas dog and Cynomolgus monkey are the most active, but neither of these latter two species is able to produce β-ZOL. Unfortunately, to our knowledge, for these two species, there is no data concerning estrogenic effects of ZEN.

Moreover, the microsomes from human females also metabolize ZEN into OH-ZEN, contrary to the microsomes from human males (13 *vs* traces pmol OH-ZEN/nmol P450/min). This last result is in agreement with the stronger activity of the isoform CYP2C8 in female human microsomal preparations than in male human microsomal preparations as indicated on the product information sheet; CYP2C8-specific hydroxylase activity was 1.8 times higher in females than in males (XenoTech LLC lot 0410044 for female and 0610050 for male). CYP2C are present in liver of all the tested animals [18–22]. As mentioned, CYP2C family recognition of substrate is subject to wide variability [15,16] explaining the different levels of ZEN metabolism. As an example despites the fact that pigs expressed CYP2C [21], these authors observed very different expression levels in the various types of pigs. This can explain the fact we were unable to measure any OH-ZEN formation in our preparations. Similarly, despite the fact that dog liver microsomes exhibit lower activities toward classical CYP2C substrates than observed for humans [22] under our conditions the former had the greatest ability to form OH-ZEN. All these results led us to conclude that several P450 isoforms can metabolize ZEN into OH-ZEN, even if CYP2C is often involved.

### 2.4. In vivo OH-ZEN occurrence in rat

Since *in vitro* formation of OH-ZEN was highest in PB-treated rat than in control animals (Table 1)*, in vivo* studies were performed in rat receiving 25 mg/kg ZEN orally. OH-ZEN was recovered in liver and in urine during the first 24 h following ZEN treatment with a maximal urinary elimination in the 3 – 6 h period (Table 3). In contrast OH-ZEN was not detected in liver of rats sacrificed at 3 h in contrast to ZEN which reach at this time a maximal value of 1.2 ± 0.2 mg/g. Maximal amounts of OH-ZEN were detected in the livers of animals sacrificed 10 hours after the ZEN treatment (Table 3). OH-ZEN was not detected in blood samples at any time of the sacrifice.

### 2.5. Estrogenic activities

Since the first studies of Boyd and Wittliff [23], various studies have confirmed that ZEN and its metabolites act as agonist ligands on both types of estrogen receptors [24]. ZEN was shown to bind and activate both ER-α and ER-β in cells transfected with human ERα and ERβ. For ERα, ZEN was found to be a full agonist and for ERβ to be a partial agonist [25]. The relative binding affinities to the rat uterine cytoplasmatic receptor for ZEN and its derivatives are α-zearalanol > α-zearalenol > β-zearalanol > ZEN > β-zearalenol [1,26]. Consequently, the species-specific rate of conversion of ZEN into α-ZOL can be regarded as a bio-activation reaction, whereas the conversion into β-ZOL can be considered as an inactivation reaction. Binding studies with subcellular fractions of various organs, including the hypothalamus, liver, uterus and the mammary gland from different species, indicated that the relative binding affinities of ZEN and its metabolites varies between 1 to 10% of that of 17β-estradiol (E_2_), with α-ZAL having a higher binding activity.

**Figure 4.**
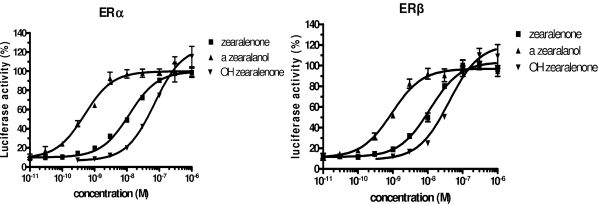
Dose-response curves of ZEN (♦), α-ZAL (▪) and OH-ZEN (▾). inhER-α (A) and hER-β (B) cells. Cell lines were incubated for 16 h at 37 °C in the presence of Zen and its metabolites at the indicated concentrations. The maximal luciferase expression was obtained with 10 nM E2. Results are esxpressed as a percentage of maximal E2 induction. Values were the mean ±SD from three separate experiments.

In order to explore whether ZEN and its metabolites (commercial α-ZAL and OH-ZEN purified from ZEN incubations with PB treated rat microsomes) could act as specific ER modulators, we used stably transfected HELN-ERs cell lines. These cells allow characterization of ER selectivity (between subtypes and species) and activity (antagonistic or agonistic) within the same cellular context [13,27]. These cells showed a slight difference sensitivity in assay for the natural ER ligand E_2_ [17]. The tests in HELN ER-α and HELN ER-β of ZEN and its metabolites yielded similar results. They behaved as full hER-α and hER-β agonists, exhibiting full dose-response curves (Figure 4).

According to the EC_50_ values, the affinity of the compounds decreased in the following order: α-ZAL > ZEN > OH-ZEN (Table 4).

ZEN and its metabolites were also tested for non-specific modulation of luciferase expression on the HELN parental cell line, which contains the same reporter gene as HELN-ERs cells but is devoid of ER. They did not showed non-specific induction of luciferase expression at concentrations lower than 1 μM in HELN cells (data not shown).

## 3. Experimental Section

### 3.1. Chemicals and Reagents

Bond Elut C18 solid-phase extraction columns were obtained from Varian (Palo Alto, CA, USA). The ZEN immunoaffinity columns (IC) ZearaStar^®^ were purchased from Romer Labs Diagnostic GMBH (Tulin, Austria). Zearalenone, zearalanone, α/β-zearalenol, α/β-zearalanol (chemical structures in Figure 1), NADPH, acetonitrile, Glucose-6-Phosphate, Glucuronidase-arylsulfatase, glycerol and ketoconazole were purchased from Sigma (St-Louis, MO, USA). All reagents were of the highest purity available.

### 3.2. Animal treatments and enzyme preparations

Male Sprague-Dawley rats (Iffa Credo, St Germain l’Arbresle, France) were housed and treated according to the French legislation in a facility authorized by the Ministry of Agriculture. Rats of initial average body weight of 200 g were used. They were fed with a standardized diet *ad libitum*. Rats were pretreated for 3 days with either saline (control) or 80 mg/kg phenobarbitone natrium salt *i.p.* in saline (PB rat) The following day they received orally 25 mg/kg zearalenone in 0.5 mL corn oil. Animals were kept in metabolic cage for urine collection following ZEN treatments. The animals were sacrificed at the indicated times and liver was taken for microsomal preparations. Post-mitochondrial (9,000 g supernatant), microsomes and cytosol fractions were prepared from liver tissue using ultracentrifugation according to the method described previously [28]. The resultant microsomal pellet was suspended in 0.1 M phosphate buffer (pH 7.4) containing 20% glycerol, the aliquots were frozen in liquid nitrogen and stored at −80 °C until use. Rat hepatocyte cultures from untreated or PBpretreated rats were performed as already described [29].

Monkeys (Cynomolgus, Macaca fascicularis), pig (Yucatan), dog (Beagle), rabbit (New Zealand), mouse (CD-1) and rat (Sprague Dawley) liver microsomes, rat 2C Supersomes^®^ were purchased from Gentest (BD Biosciences, NJ, USA). Pools of human livers for female or male were purchased from Xenotech (Tebu-Bio, Le Perray en Yvelines, France). Human CYP Bactosomes^®^ were provided by Cypex (Tebu-Bio, Le Perray en Yvelines, France).

### 3.3. Incubations with zearalenone

Human expressed CYPs or microsomal solutions (1 μM P450 final concentration) were incubated with a NADPH generating system (1 mM NADP + 10 mM Glucose-6-Phosphate, 1 IU G_6_PDH), 100 μM MgCl_2_ and 50 μM zearalenone for the given times at 37 °C in 200 μL 0.1 M phosphate buffer (pH 7.4). Control incubations without microsomes, or without the NADPH generating system, were performed in parallel. Incubations were stopped by addition of the same volume of cold acetonitrile and stored for a few minutes at −20 °C. After centrifugation at 7,500 g for 10 minutes, the supernatants were stored at −20 °C until analysis.

Incubations with hepatocytes were performed 24 h after plating in the presence of phosphate buffer and 10 or 20 μM ZEN for 2 or 4 h. Incubations were stopped upon addition of cold acétonitrile, thawing followed by centrifugation before HPLC-MS analysis.

### 3.4. HPLC Sample preparations

The extraction procedure of ZEN and its metabolites were adapted from [30].

#### 3.4.1. Urine sample

Rat urine (1 mL) was mixed with 5 mL of 50 mM ammonium acetate buffer, pH 4.8. This solution was incubated for 15 h at 37 °C with 20 μL of glucuronidase/arylsulfatase solution. After adjusting the pH with glacial acetic acid to pH 4 the solution was purified on a 100 mg Sep-Pac^®^ C18 solid-phase extraction column and eluted with 1.5 mL of MeOH. This elute was mixed with 20 mL of phosphate buffer saline, pH 7.4, and loaded on to an immunoaffinity column, washed with 15 mL of water, and eluted with 1.5 mL of ACN. After evaporation under vacuum, the residue was reconstituted in 150 μL of the HPLC mobile phase. A 20 μL amount of this solution was injected into the HPLC-MS system for analysis. Recovery of ZEN was controlled using samples spiked with known amounts. Similar treatments were performed for plasma or 9,000 g supernatant from liver phosphate buffered homogenate of rat treated or by zearalenone.

### 3.5. HPLC and mass spectrometry analysis

The HPLC system used in LC-MS consisted of a LC 1100 system (Agilent, CA, US) coupled with an Ion Trap Mass spectrometer (Bruker Daltonics Esquire HCT) using an ESI interface operating in negative mode (Bruker Daltonics, MA, USA) as already described [31].

### 3.6. Absolute and relative recoveries, precision, method detection limits (MDL) and linearity

Before quantifying the concentration of ZEN and its metabolites in biological samples standard curves were determined using solutions for each corresponding matrix. The relative recovery was defined as the ratio between the quantified and the spiked amount. The precision of the analytical method was defined as the mean relative standard deviation of five replicates at the chosen concentration level. For all analyses, the MDL was defined as three times the mean blank level. Linearity was estimated from calibration curve performed with ZEN.

For OH-ZEN UV quantification at 280 nm, we assumed, based on the identity of the aromatic part of ZEN and OH-ZEN structures, that both compounds have the same extinction coefficient. The presence of the coupled on-line UV_280 nm_ and MS detection system allowed us to verify that for each analytical series that the UV/MS ratio was virtually constant.

### 3.7. Estrogenic activities

The stably transfected HELN-ER-α and -β cell lines were obtained as previously described [13,27]. Briefly, generation of HELN-ER-α and -ER-β reporter cell lines was performed in two steps. The estrogen responsive reporter gene was first stably transfected into HeLa cells, generating a HELN cell line and, in a second step, these HELN cells were transfected with –ER-α, -ER-β plasmid constructs to obtain the HELN-ER alpha and -ER beta cell lines, respectively. HELN-ERs cells were cultured in DMEM F12 without phenol red, supplemented with 6% dextran-coated charcoal (DCC)-treated FCS (6% DCC-FCS), 1% antibiotic, 1 mg/mL G418 and 0.5 μg/mL puromycin.

Reporter cells were seeded in white opaque 96-well tissue culture plates at a density of 50,000 cells per well. Each well contained 150 μL of test culture medium. The compounds to be tested were 4-fold concentrated in the same medium and 50 μL was added per well 8 h after seeding. Cell lines were incubated for 16 h with the compounds at 37 °C. At the end of incubation, the medium containing test compounds was removed and replaced by test culture medium containing 0.3 mM luciferin. The 96-well plate was then introduced in a Microbeta Wallac luminometer (Centro LB 960, Berthold Technologies) and luminescence was measured in intact living cells for 2 s.

To study hER α and hER β agonistic activities, HELN ERs were tested in the presence of increasing concentrations (0.01 nM – 1 μM) of the compounds under investigation. The tests were performed three times in quadruplicate for each concentration. The results were expressed as a percentage of the maximal luciferase activity. Maximal luciferase activity (100 %) was obtained in the presence of 10 nM estradiol. For each compound, the estrogenic potency corresponding to the concentration yielding half-maximal luciferase activity (EC_50_ value) was calculated. Compounds were also tested for nonspecific modulation of luciferase expression on the HELN parental cell line, which contains the same reporter gene as HELN-ERs cells but is devoid of ER.

#### 3.7.1. Data analysis

In the trans-activation assay, each compound was tested at various concentrations in at least three independent experiments. For each experiment, tests were performed in quadruplicate for each concentration and data were expressed as mean ± SD. Data were analysed for significant differences using one-way ANOVA followed by Dunnet’s post comparison test (*versus* control). Individual doseresponse curves, in the absence and in the presence of agonist, were fitted using the sigmoidal doseresponse function of a graphics and statistics software (Graph-Pad Prism, version 4.0, 2003, Graphpad Software Incorporated, San Diego. CA). Transactivation data are presented as EC_50_ values, effective concentration for half-maximal luciferase activity and as IC_50_ values, half-maximal inhibitory concentration for each compound tested.

## 4. Conclusions

Until this present work, most studies on ZEN metabolism have dealt with the analysis and/or quantification of α/β-ZOL, α/β-ZAL or ZAN. Oxidative metabolism of ZEN to its hydroxylated metabolite is one other possible metabolic pathway. This compound can be detected *in vivo* after rat treatment and can be formed in liver of different animal species including human. The formation of the hydroxylated metabolite is catalysed by several isoforms of cytochromes P450. Only one recent study [11] revealed the formation of hydroxylated ZEN metabolites. Here we have highlighted one of these OH-ZEN forms and studied its estrogenic activity. The OH-ZEN estrogenic activities were revealed to be limited and not more important than either ZEN or α-ZAL. Nevertheless, it has a more important activity than any other hydroxy metabolite of ZEN, formed directly by the micro-organism and previously studied by El Sharkawy and co-workers [32,33].

## Figures and Tables

**Figure 3.**
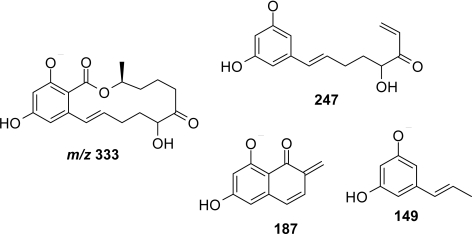
Structure of the major OH-ZEN deduced from its fragmentation. Only the fragments allowing the precise location of the oxidation site are given.

**Table 1.** Metabolism of ZEN using expressed human or rat cytochromes P450.

	OH-ZEN		OH-ZEN
H CYP1A1	n.d.	H CYP1A2	n.d.
H CYP2A6	n.d.	H CYP2B6	n.d.
H CYP2C8	2.75	H CYP2C9	n.d.
H CYP2C18	n.d.	H CYP2C19	n.d.
H CYP2D6	n.d.	H CYP2E1	n.d.
H CYP3A4	2.20	H CYP3A5	2.81
R CYP2C6	0.36	R CYP2C11	2.40
R CYP2C12	n.d;	R CYP2C13	0.54

The amount of OH-ZEN was determined by analysis of the absorbance at 280 nm and confirmed by HPLC-MS. The results are expressed as OH-ZEN formation (nmole/nmole P450) after 60 minutes incubation of ZEN with 1 μM human (H) or rat (R) expressed P450s (n.d. : not detectable; n = 2).

**Table 2.** *In vitro* metabolism of zearalenone using human and animal liver microsomal preparations.

	α-ZOL	β-ZOL	OH-ZEN
Mouse	41.3 ± 11.5	n.d.	n.d.
Rat	14.7 ± 2.5	n.d.	1.7 ± 0.7
PB-treated rat	n.d.	3.3 ± 0.4	4.9 ± 0.3
Rabbit	2.5 ± 0.4	1.9 ± 0.4	2.2±0.2
Dog	2.8 ± 0.3	n.d.	8.1±0.5
Pig	7.4 ± 0.6	10.0 ± 1.8	n.d.
Cynomolgus monkey	4.0 ± 0.4	n.d.	8.4±4.0
Human Female	3.3 ± 0.5	0.3 ± 0.1	0.4±0.1
Human Male	3.6 ± 0.3	n.d.	traces

The formation rates of ZEN metabolites : α-, β-ZOL and OH-ZEN were determined after 30 min incubation using 1 μM total cytochrome P450 in the presence of 50 μM ZEN and a NADPH-generating system in 0.1 M phosphate buffer, pH 7.4. The results (n = 4) are expressed as nmoles/30 min/nmole P450 from HPLC-UV detection at 280 nm. n.d. means not detectable and traces means detected, but under the level of quantification.

**Table 3.** Amounts of OH-ZEN recovered in liver and in urine of control rats and PBpretreated rats and rats treated orally with 25 mg/kg ZEN.

Liver	OH-ZEN μg/g	Urine Collection times	OH-ZEN μg/mL
3 h	0	0-3 h	0.9
6 h	17 ± 2	3–6 h	2.0
10 h	33.5 ± 4.5	6–10h	1.1
24 h	14.2 ± 1.3	10–24 h	0.4

The quantity of OH-ZEN was determined by UV absorption at 280 nm and identity was confirmed by HPLC-MS. The results are the means of two determinations performed on 9,000 g supernatant of 3 livers treated individually and on pooled urines of three animals.

**Table 4.** EC_50_ values for ZEN, α-ZAL, OH-ZEN for ER-α and ER-β.

EC_50_	E2	α–ZAL	ZEN	OH-ZEN
ER-α	0.019± 0.005 nM	0.49 ± 00.9 nM	11.78 ± 1.9 nM	70.31 ± 15.55 nM
ER-β	0.067± 0.007 nM	0.93 ± 0.2 nM	11.46 ± 2.5 nM	40.27 ± 21.89 nM

Effective concentrations for half-maximal luciferase activity (EC50) of E2, ZEN and its metabolites on transcriptional activation through ER α and ER β.
